# Fluoroquinolone-Resistant Group B Streptococci in Acute Exacerbation of Chronic Bronchitis

**DOI:** 10.3201/eid1402.071006

**Published:** 2008-02

**Authors:** Asmaa Tazi, Thomas Gueudet, Emanuelle Varon, Liliane Gilly, Patrick Trieu-Cuot, Claire Poyart

**Affiliations:** * Assistance Publique-Hôpitaux de Paris, Paris, France; † Institut National de la Santé de la Recherche Médicale, Paris, France; ‡ Université Paris Descartes, Paris, France; § Laboratoire Schuh-Biosphere, Strasbourg, France; ¶ Institut Pasteur, Paris, France

**Keywords:** Streptococcus agalactiae, fluoroquinolone, group B streptococci, drug resistance, antibiotics, bronchitis, letter

**To the Editor:** Fluoroquinolones (FQs) that are active against streptococcal species (e.g., levofloxacin and moxifloxacin) have been recommended by numerous national health authorities and international organizations for treating acute exacerbations of chronic bronchitis and pneumonia in adults (*1*). However, use of these antimicrobial drugs for treating community-acquired infections has led to an increase in FQ-resistant strains in bacteria such as *Streptococcus pneumoniae*. Group B streptococci (GBS, e.g., *S*. *agalactiae*) are the leading cause of invasive infections (pneumonia, septicemia, and meningitis) in neonates. GBS are also associated with bacteremia, endocarditis, and arthritis, and are responsible for deaths and illness in nonpregnant women with underlying diseases and in elderly adults (*2*). We describe, to our knowledge, the first GBS clinical isolate in France resistant to FQ; the isolate was from a patient treated with levofloxacin.

GBS CNR0717 strain was isolated as the predominant bacterium in a culture (>10^7^ CFU/mL) from 2 purulent sputum samples from an 80-year-old man (leukocytes >25, epithelial cells <10) obtained 8 days apart. This patient was treated for 2 weeks with levofloxacin, 750 mg/day, for acute exacerbation of chronic bronchitis. No other relevant respiratory bacterial pathogens were present in these samples. GBS CNR0717, a capsular serotype IV strain, was suspected to have reduced susceptibility to FQs because no inhibition zone was observed around disks containing norfloxacin and pefloxacin disks, and reduced diameters were observed around disks containing ciprofloxacin and levofloxacin. Antibiograms were performed according to recommendations of the Clinical and Laboratory Standards Institute (*3*) on Mueller Hinton agar (Bio-Rad, Marnes la Coquette, France) supplemented with 5% horse blood. This strain was susceptible to all other antimicrobial drugs usually active against GBS (penicillin, erythromycin, clindamycin, tetracycline, rifampicin, vancomycin) and showed low-level resistance against aminoglycosides. MICs for 6 FQs (Table) indicate that GBS CNR0717 was highly resistant to pefloxacin and norfloxacin, with MICs >64 mg/L, and showed increased MICs for ciprofloxacin, sparfloxacin, levofloxacin, and moxifloxacin. No reduction of FQ MICs was observed with reserpine (10 mg/L), which indicated that resistance to FQ was not caused by an active efflux pump system.

**Table:** MICs of fluoroquinolones for strains of group B streptococci (GBS), France

Strain	MIC (mg/L)*					
	Pef	Nor	Cip	Spa	Lev	Mox
GBS CNR07017	>64	>64	4	1	4	1
GBS NEM316	16	8	2	0.5	1	0.25

*Pef, pefloxacin; Nor, norfloxacin; Cip, ciprofloxacin; Spa, sparfloxacin; Lev, levofloxacin; Mox, moxifloxacin.

Three major mutations have been reported for FQ resistance in streptococci at codon positions 81 in *gyrA* and 79 or 83 in *parC* (*4*). DNA sequence analysis of these regions showed a mutation in *parC* (Ser 79 → Tyr) but not in the wild-type susceptible strain (NEM316). No mutation was detected in the *gyrA* gene. FQ resistance in streptococci is acquired through a stepwise process and has been extensively studied in *S*. *pneumoniae*. First-step mutants conferring low-level resistance generally result from mutations in either *gyrA* or *parC*. There is also a molecule-dependent target specificity: mutations in *parC* are generally selected by pefloxacin, ciprofloxacin, and levofloxacin, and those in *gyrA* are selected by sparfloxacin, gatifloxacin, moxifloxacin, gemifloxacin, and garenoxacin (*5*). In second-step mutants, mutations are present in both *parC* and *gyrA* and confer resistance to the antistreptococcal FQs levofloxacin, moxifloxacin, and gatifloxacin.

FQ resistance in GBS has been reported in Japan, the United States, and Spain (*6*–*8*). Up to now, all FQ-resistant GBS strains described were highly resistant because of point mutations in *gyrA* and *parC* QRDR; a *parC* mutation at position 79 was present in all strains. These strains were isolated from elderly adults who, in some cases, had received quinolone therapy. Low-level resistance to FQ in GBS CNR0717 was associated with a Ser 79 → Tyr mutation in *parC*. Therefore, although the FQ sensitivity of this strain is unknown, a first-step mutant could have been selected in vivo as our patient was treated with levofloxacin for 2 weeks.

GBS is an unusual cause of acute bacterial exacerbation of chronic bronchitis compared with other respiratory pathogens such as *S*. *pneumoniae,* but pathologies associated with this bacterium are changing. Clinical microbiologists should be aware of these changes and test isolates of *Streptococcus* spp. for susceptibility to FQs. This report indicates that FQ resistance among streptococci is a growing concern and that levofloxacin can select in vivo *parC* first-step mutants that will facilitate emergence of high-level FQ-resistant GBS strains, as demonstrated in vitro for *S*. *pneumoniae* (*9*). Finally, although FQ treatment is recommended for high-risk groups with acute exacerbations of chronic bronchitis, these antimicrobial drugs must be reserved for situations in which there are no effective alternative drugs to treat infections caused by multidrug-resistant bacteria. For susceptible strains, β-lactams, which still constitute the first-line recommended antimicrobial drugs, should be used for treatment of these patients (*10*).
